# Rosiglitazone reduces breast cancer risk in Taiwanese female patients with type 2 diabetes mellitus

**DOI:** 10.18632/oncotarget.13824

**Published:** 2016-12-08

**Authors:** Chin-Hsiao Tseng

**Affiliations:** ^1^ Department of Internal Medicine, National Taiwan University College of Medicine, Taipei, Taiwan; ^2^ Division of Endocrinology and Metabolism, Department of Internal Medicine, National Taiwan University Hospital, Taipei, Taiwan; ^3^ Division of Environmental Health and Occupational Medicine of the National Health Research Institutes, Zhunan, Taiwan

**Keywords:** breast cancer, diabetes mellitus, rosiglitazone, Taiwan

## Abstract

This study investigated whether rosiglitazone may affect breast cancer risk in female patients with type 2 diabetes mellitus in Taiwan. The reimbursement database of all female patients with type 2 diabetes mellitus under oral antidiabetic agents or insulin from 1996 to 2009 was retrieved from the National Health Insurance. An entry date was set on 1 January 2006 and a total of 431447 patients were followed up for breast cancer incidence till the end of 2009. Incidences for ever users, never users and subgroups of rosiglitazone dose-response parameters (tertile cutoffs of cumulative duration and cumulative dose) were calculated and hazard ratios estimated by Cox regression. There were 53029 ever users and 378418 never users, respective numbers of incident breast cancer 410 (0.77%) and 3292 (0.87%), and respective incidence 217.53 and 249.12 per 100000 person-years. The overall hazard ratio was 0.889 (95% confidence interval: 0.797−0.992) in the fully adjusted model. Significantly lower risk was observed for the third tertiles of cumulative duration (> 14 months) and cumulative dose (> 1792 mg) while compared to never users, with respective adjusted hazard ratio of 0.815 (95% confidence interval: 0.682−0.973) and 0.815 (95% confidence interval: 0.682−0.974). Additionally, a significant interaction between metformin and rosiglitazone was observed. The lowest risk was seen in patients who used both drugs (hazard ratio 0.812, 95% confidence interval: 0.705−0.934). In conclusion, rosiglitazone reduces breast cancer risk in female patients with type 2 diabetes mellitus, which shows a significant interaction with metformin.

## INTRODUCTION

Rosiglitazone shows antiproliferative and apoptotic actions on breast cancer cells; and it may induce autophagy and inhibit the invasiveness and metastasis in breast cancer cell lines [1, 2]. In rats treated with the carcinogen 7,12-dimethylbenz(a)anthracene, rosiglitazone suppresses mammary tumor growth [3]. A recent study found that rosiglitazone may inhibit breast cancer growth in mice by suppressing the expression of a pro-inflammatory and pro-tumor protein Gpr132 in tumor-associated macrophages [4].

Whether these beneficial effects of rosiglitazone on breast cancer observed in cellular and animal studies can be extended to diabetic patients who use rosiglitazone as a therapeutic agent remains to be explored. In a pilot short-term clinical study conducted in women with newly diagnosed early breast cancer (stage 0–II), treatment with rosiglitazone 4 mg twice daily for 2–6 weeks did not show promising anticancer effect [5]. Similarly, a meta-analysis including 80 randomized clinical trials of rosiglitazone treatment > 24 weeks did not show a beneficial effect [6]. The summary hazard ratio for incidence of mammary and/or female genital tract malignancies associated with rosiglitazone was 1.19 (95% confidence interval: 0.62-2.26) [6]. On the other hand, an observational cohort study conducted in France suggested a significantly lower risk of breast cancer associated with rosiglitazone use, with an adjusted hazard ratio of 0.80 (95% confidence interval: 0.73-0.88) [7].

The effects of rosiglitazone and pioglitazone on breast cancer may differ as shown in *in vitro* and *in vivo* studies [8]; and in human observational studies [7, 9]. For examples, rosiglitazone but not pioglitazone may induce the expression of protein phosphatase and tensin homolog located on chromosome ten, a tumor suppressor gene that may play some role in the pathogenesis of breast cancer [8]. The French study conducted in humans also showed a significantly lower risk associated with rosiglitazone but not with pioglitazone [7]. However, this French study aimed primarily at analyzing the cancer risk associated with pioglitazone and has several limitations such as a restriction of the patients’ age within 40–79 years and being able to adjust only for age and other antidiabetic drugs. The investigators did not consider other potential confounders that have been recognized as important risk factors of breast cancer, such as benign breast conditions [10] and use of estrogen [11], and the protective effect of aspirin [12]. Furthermore, the French study failed to consider the effect of differential detection examinations that had been conducted in patients with and without the use rosiglitazone.

In the present study, the association between rosiglitazone and breast cancer was investigated in the Taiwanese female patients with type 2 diabetes mellitus by using the reimbursement database of the National Health Insurance (NHI) from the whole population during the period from 1996 to 2009.

## RESULTS

Table 1 compares the baseline characteristics between ever users (*n* = 53029) and never users (*n* = 378418) of rosiglitazone. All variables differed significantly. Ever users are characterized by older age, higher proportion with a diabetes duration ≥ 5 years, higher proportions of all comorbidities and other cancer, higher proportions of using other medications, and a higher proportion of receiving potential detection examinations.

**Table 1:** Baseline characteristics between never users and ever users of rosiglitazone

Variables	Never users		Ever users		*P*
	*n*	%	*n*	%	(Chi-square test)
*n* = 431447	378418		53029		
Age (years)					
< 40	13108	3.46	1240	2.34	< 0.0001
40–49	37768	9.98	4495	8.48	
50–59	88584	23.41	12855	24.24	
60–69	105895	27.98	16598	31.30	
≥ 70	133063	35.16	17841	33.64	
Diabetes duration (years)					
< 1	30516	8.06	568	1.07	< 0.0001
1–3	58926	15.57	3002	5.66	
3–4	56427	14.91	4902	9.24	
≥ 5	232549	61.45	44557	84.02	
Hypertension	225631	59.62	41722	78.68	< 0.0001
Chronic obstructive pulmonary disease	54417	14.38	12636	23.83	< 0.0001
Cerebrovascular disease	58930	15.57	14042	26.48	< 0.0001
Nephropathy	45488	12.02	12238	23.08	< 0.0001
Ischemic heart disease	89320	23.60	22117	41.71	< 0.0001
Peripheral arterial disease	44918	11.87	13274	25.03	< 0.0001
Eye disease	31704	8.38	11814	22.28	< 0.0001
Obesity	6672	1.76	1238	2.33	< 0.0001
Dyslipidemia	187423	49.53	36600	69.02	< 0.0001
Benign breast conditions	2463	0.65	471	0.89	< 0.0001
Other cancer prior to baseline	42025	11.11	6351	11.98	< 0.0001
Sulfonylurea	272445	72.00	51200	96.55	< 0.0001
Metformin	246129	65.04	50145	94.56	< 0.0001
Acarbose	39774	10.51	20852	39.32	< 0.0001
Insulin	50786	13.42	21237	40.05	< 0.0001
Aspirin	113456	29.98	28240	53.25	< 0.0001
Estrogen	30722	8.12	7968	15.03	< 0.0001
Potential detection examinations	5333	1.41	971	1.83	< 0.0001

Table 2 shows the incidences of breast cancer in never users, ever users and different subgroups of the dose-response parameters (i.e., the tertile cutoffs of cumulative duration and cumulative dose) of rosiglitazone. The incidence rate in never users and ever users was 249.12 and 217.53 per 100,000 person-years, respectively.

**Table 2:** Incidence of breast cancer by rosiglitazone exposure at entry

Rosiglitazone use	Case number	Incident breast cancer	%	Person-years	Incidence rate
					(per 100,000 person-years)
Never users	378418	3292	0.87	1321464.67	249.12
Ever users	53029	410	0.77	188477.25	217.53
**Cumulative duration (months)**					
Never users	378418	3292	0.87	1321464.67	249.12
< 3.73	16861	138	0.82	58187.58	237.16
3.73–14	17901	138	0.77	63472.08	217.42
x> 14	18267	134	0.73	66817.58	200.55
**Cumulative dose (mg)**					
Never users	378418	3292	0.87	1321464.67	249.12
< 448	16845	136	0.81	58281.33	233.35
448–1792	18037	141	0.78	63910.75	220.62
> 1792	18147	133	0.73	66285.17	200.65

Table 3 shows the hazard ratios with regards to rosiglitazone exposure in different models. The overall hazard ratios for ever users versus never users showed a significantly lower risk in all models. While evaluating the dose-response relationship, a lower risk was observed in the third tertiles of both cumulative duration and cumulative dose in all models and all *P* values for the trend were significant.

**Table 3:** Rosiglitazone exposure at entry and hazard ratios for breast cancer in women with type 2 diabetes mellitus

Rosiglitazone use	Model I			Model II			Model III			Model IV		
	HR	95% CI	*P* value	HR	95% CI	*P* value	HR	95% CI	*P* value	HR	95% CI	*P* value
Ever users vs. never users	0.854	(0.771, 0.946)	0.0026	0.855	(0.772, 0.948)	0.0028	0.869	(0.783, 0.964)	0.0081	0.889	(0.797, 0.992)	0.0359
**Cumulative duration (months)**									
< 3.73 vs. never users	0.945	(0.797, 1.121)	0.5168	0.945	(0.797, 1.121)	0.5159	0.966	(0.814, 1.146)	0.6904	0.975	(0.818, 1.161)	0.7723
3.73–14 vs. never users	0.838	(0.707, 0.994)	0.0420	0.838	(0.707, 0.994)	0.0422	0.860	(0.725, 1.021)	0.0858	0.889	(0.746, 1.059)	0.1862
> 14 vs. never users	0.791	(0.666, 0.941)	0.0079	0.794	(0.668, 0.944)	0.0090	0.795	(0.668, 0.945)	0.0094	0.815	(0.682, 0.973)	0.0239
*P*-trend			0.0010			0.0011			0.0025			0.0127
**Cumulative dose (mg)**											
< 448 vs. never users	0.926	(0.780, 1.100)	0.3814	0.926	(0.780, 1.100)	0.3816	0.947	(0.797, 1.124)	0.5318	0.908	(0.762, 1.083)	0.2837
448–1792 vs. never users	0.853	(0.720, 1.009)	0.0637	0.853	(0.721, 1.010)	0.0652	0.877	(0.740, 1.039)	0.1281	0.950	(0.799, 1.130)	0.5621
> 1792 vs. never users	0.793	(0.666, 0.943)	0.0086	0.795	(0.668, 0.945)	0.0095	0.795	(0.668, 0.946)	0.0097	0.815	(0.682, 0.974)	0.0246
*P*-trend			0.0012			0.0014			0.0030			0.0236

HR: hazard ratio; CI: confidence interval

Model I: unadjusted; Model II: adjusted for age; Model III: adjusted for age, benign breast conditions, use of estrogen and use of aspirin; Model IV: adjusted for all variables in Table 1.

Table 4 shows the joint effects of metformin and rosiglitazone on breast cancer risk. There was a significant interaction between these two drugs. While compared to patients who had not been treated with either drug, users of metformin without rosiglitazone showed a significant 10% risk reduction, and users of rosiglitazone without metformin showed a non-significant 16% risk reduction. The lowest risk was observed in users of both drugs (hazard ratio 0.812, 95% confidence interval: 0.705-0.934).

**Table 4:** Joint effects of metformin and rosiglitazone on breast cancer risk

Metformin/Rosiglitazone use	Case number	Incident breast cancer	%	Person-years	Incidence rate (per 100,000 person-year)	Hazard ratio	95% Confidence interval	*P* value
Metformin (−) / Rosiglitazone (−)	132613	1264	0.95	460185.58	274.67	1.000		
Metformin (+) / Rosiglitazone (−)	245805	2028	0.83	861279.08	235.46	0.900	(0.817–0.991)	0.0323
Metformin (−) / Rosiglitazone (+)	2912	24	0.82	10044.33	238.94	0.836	(0.557–1.255)	0.3872
Metformin (+) / Rosiglitazone (+)	50117	386	0.77	178432.92	216.33	0.812	(0.705–0.934)	0.0036
*P*-interaction								0.0434

Hazard ratios are adjusted for all variables in Table 1.

## DISCUSSION

This is the first study conducted in an Asian population showing that rosiglitazone reduces the risk of breast cancer in Taiwanese female patients with type 2 diabetes mellitus. The findings were consistent in all models and showed an inverse dose-response relationship with both cumulative duration and cumulative dose of therapy (Table 3). The reduced risk was statistically significant when the cumulative duration was > 14 months or when the cumulative dose was > 1792 mg (Table 3). Furthermore, a significant interaction between metformin and rosiglitazone could be observed (Table 4). The lowest risk was seen in patients who used both metformin and rosiglitazone (Table 4).

Such a promising effect provided a rationale for more in-depth investigation on rosiglitazone in the prevention and treatment of breast cancer. Because previous *in vitro* and *in vivo* studies [8] and human observational studies [7, 9] suggested that rosiglitazone and pioglitazone may act differently in breast cancer and we have excluded pioglitazone from analyses, whether the beneficial effect of rosiglitazone can be extended to the other thiazolidinediones remains to be answered. Furthermore, the results could not be generalized to nondiabetic individuals, because only patients with type 2 diabetes mellitus were analyzed. Because rosiglitazone rarely induces hypoglycemia when used alone, it will be worthwhile to investigate its effects in nondiabetic women.

Patients with type 2 diabetes mellitus are characterized by insulin resistance [13, 14], and female patients with type 2 diabetes mellitus are at an increased risk of breast cancer [15]. Studies suggested that insulin resistance and hyperinsulinemia may play an important role on the development of breast cancer [16, 17]. Adiponectin and leptin are two adipokines that may act oppositely on the risk of breast cancer [18]. While adiponectin may reduce the risk, leptin, on the other hand, is potentially associated with a higher risk [18]. Rosiglitazone and pioglitazone have both been shown to increase the level of adiponectin with little effect on leptin [19, 20]. Therefore, a possible explanation for the beneficial effect of rosiglitazone on breast cancer is through its action on the improvement of insulin resistance by elevating adiponectin levels. However, because both rosiglitazone and pioglitazone increase adiponectin and improve insulin resistance, but only rosiglitazone and probably not pioglitazone may show a beneficial effect on breast cancer risk [7], this may argue against such a mechanism. On the other hand, rosiglitazone may induce the expression of a tumor suppressor gene related to breast cancer but pioglitazone may not [8]. This could partially explain the different clinical effects of rosiglitazone and pioglitazone on breast cancer. The recent study by Cheng et al. pointed to another potential mechanism of rosiglitazone on breast cancer development and growth via the inhibition of a pro-inflammatory and pro-tumor protein Gpr132 in tumor-associated macrophages [4]. Because the effect of pioglitazone on Gpr132 expression has not been examined, it remains unknown whether different drugs in the class of thiazolidinediones may exert different effects on breast cancer risk through their discrepant effects on Gpr132.

The present study confirmed our previous observation of a beneficial effect of metformin on breast cancer [21] (Table 4). Additionally, the present study revealed a significant interaction between metformin and rosiglitazone and the lowest risk was observed in users of both drugs (Table 4).

Because our previous studies suggested that use of insulin [22, 23] and/or sulfonylurea [24] are associated with a significantly higher risk of breast cancer, the higher proportions of their use in ever users of rosiglitazone (Table 1) might have only underestimated the beneficial effect of rosiglitazone.

This study has several strengths. First, potential risk of selection bias related to sampling error was unlikely because the database was derived from the whole population and it covered the whole period from the beginning of the marketing of rosiglitazone from 2001 in Taiwan to the end of 2009. Second, because the diagnosis of breast cancer was captured from all claim records from outpatient visits and hospital admission, the ascertainment rate should be high. Third, detection rate of breast cancer is less likely to be affected by socioeconomic status because most medical co-payments can be waived in patients with a diagnosis of cancer and there is a low drug cost-sharing in patients with low-income, in veterans or in patients with prescription refills for chronic disease. Fourth, the use of medical records significantly reduced the bias related to self-reporting.

The study may also suffer from some limitations. First, there was a lack of measurement data for potential confounders such as family history, lifestyle, diet, obesity, tobacco smoking, alcohol drinking, and genetic parameters. Second, no biochemical data were available for evaluating their impacts. These may include hormonal profiles, blood glucose, hemoglobin A_1C_, insulin, C-peptide, or parameters for insulin resistance. Third, we did not have information on the pathology, grading and staging of breast cancer for more in-depth analyses. Fourth, misclassification of breast cancer might occur in some patients. However, such a probability might not be high because mislabeling of a cancer diagnosis in the prescription handed to the patients would not be acceptable when the patients saw the diagnosis.

In summary, this study supports a beneficial effect of rosiglitazone on the prevention of breast cancer in Taiwanese female patients with type 2 diabetes mellitus. Furthermore, a significant interaction between metformin and rosiglitazone can be observed.

## MATERIALS AND METHODS

The study was approved by an ethic review board of the National Health Research Institutes (approval number 99274).

In Taiwan, a compulsory and universal health care system (the so-called NHI) was implemented since March 1995. More than 99% of the residents are covered by the NHI, and > 98% of the hospitals throughout Taiwan are under contract with the NHI. Computerized and standard claim documents should be submitted by the contracted medical institutes for reimbursement.

The NHI reimbursement database, which contains detailed records on every visit (outpatient visits and hospital admission) for each patient, is handled by the National Health Research Institutes for academic research. The records include principal and secondary diagnostic codes, prescription orders, and claimed expenses.

Personal information of each individual was deidentified for the protection of privacy. Diabetes was coded 250.XX and breast cancer 174, based on the *International Classification of Diseases, Ninth Revision, Clinical Modification* (ICD-9-CM).

The database of all patients ever diagnosed of diabetes and under treatment with either oral antidiabetic agents or insulin during 1996–2009 was retrieved from the whole nation. Because pioglitazone may increase the risk of bladder cancer [25–29], patients who had been treated with pioglitazone (*n* = 235287) were first excluded to avoid its contamination. An entry date was set on 1 January 2006. After excluding male patients (*n* = 935445), patients with a diagnosis of diabetes after 2006 (*n* = 342351), patients holding a Severe Morbidity Card with a diagnosis of type 1 diabetes (*n* = 7120, in Taiwan, a so-called “Severe Morbidity Card” can be issued to patients with type 1 diabetes after certified diagnosis and the patients can be waived of much of the medical co-payments), patients diagnosed of breast cancer before 2006 (*n* = 14755), patients who died (*n* = 96320) or withdrew from the NHI (*n* = 12502) before entry date, duplicated identification number (*n* = 106), and unclear information on date of birth or sex (*n* = 5120), a total of 431447 female patients diagnosed of type 2 diabetes mellitus and under treatment with oral antidiabetic agents or insulin were included into the analyses.

Patients ever prescribed rosiglitazone before entry date were defined as ever users; and those who had never been treated with rosiglitazone before entry date were defined as never users. The tertile cutoffs of cumulative duration of therapy in months and cumulative dose in mg were calculated and used as indicators of a dose-response relationship.

An entry date set at the beginning of 2006 was based on the following reasons: 1) Rosiglitazone was marketed in 2001 in Taiwan, and this entry date provided a longest exposure to rosiglitazone for 4 to 5 years at entry and a longest follow-up duration of 4 years; and 2) Pioglitazone was noted to potentially increase the risk of bladder cancer in the PROspective pioglitAzone Clinical Trial In macroVascular Events in 2005 [26], and in 2007, an increased risk of acute myocardial infarction has been challenged with the use of rosiglitazone [30]. These reports had caused tremendous changes in the physicians’ prescription behavior and the patients’ adherence. The physicians would tend to withdraw thiazolidinediones including rosiglitazone and pioglitazone (troglitazone was not available in Taiwan) and the patients might not adhere to the drugs even if they were prescribed. Therefore, a later entry date after the year 2006 would not only make the estimation of the cumulative duration and cumulative dose of rosiglitazone less reliable, it could also shorten the follow-up duration for cancer incidence.

All comorbidities and covariates were determined as a status/diagnosis before the entry date. The ICD-9-CM codes for the comorbidities were [31–35]: nephropathy 580-589, hypertension 401-405, chronic obstructive pulmonary disease (a surrogate for smoking) 490-496, cerebrovascular disease 430-438, ischemic heart disease 410-414, peripheral arterial disease 250.7, 785.4, 443.81 and 440-448, eye disease 250.5, 362.0, 369, 366.41 and 365.44, obesity 278, dyslipidemia 272.0-272.4, benign breast conditions 217, 610, 611, 612, 675, 676, and cancer other than breast cancer 140-208 (excluding 174). Medications included sulfonylurea, metformin, insulin, acarbose, aspirin and estrogen. The following examinations were considered as “potential detection examinations” that might lead to a confounding effect: 1) mammography; and/or 2) breast sonography. Baseline characteristics were compared by Chi-square test between ever users and never users of rosiglitazone.

The incidence density of breast cancer was calculated for different subgroups of rosiglitazone exposure including ever users, never users and the tertiles of cumulative duration and cumulative dose. The numerator was the number of patients with incident breast cancer during follow-up, and the denominator was the person-years of follow-up. For ever users, the follow-up ended on the date of breast cancer diagnosis or on the date of the last record in the reimbursement database in individuals without incident breast cancer. For never users, the follow-up ended on the date of breast cancer diagnosis, or on the date of rosiglitazone initiation or the last reimbursement record, depending on which occurring first. This would ensure no exposure to rosiglitazone at the end of follow-up in the never users.

Cox proportional hazards regression was used to estimate the hazard ratios for breast cancer for ever users and for the tertiles of cumulative duration and cumulative dose, using never users as the referent group. The following four models were created: 1) unadjusted; 2) adjusted for age; 3) adjusted for selected important risk factors of breast cancer including age, benign breast conditions, use of estrogen, and use of aspirin; and 4) adjusted for all baseline characteristics (fully adjusted).

Metformin has been shown to reduce the risk of various types of cancer [36–42]. Because our previous study also suggested that metformin may prevent breast cancer [21], additional analyses were conducted to evaluate the potential joint effects of metformin and rosiglitazone. The incidence rates of breast cancer and the fully adjusted hazard ratios were calculated for the following 4 subgroups with regards to the use of metformin and rosiglitazone: 1) metformin (−)/rosiglitazone (−) as referent, 2) metformin (+)/rosiglitazone (−), 3) metformin (−)/rosiglitazone (+), and 4) metformin (+)/rosiglitazone (+).

Analyses were conducted using SAS statistical software, version 9.3 (SAS Institute, Cary, NC). *P* < 0.05 was considered statistically significant.
